# “Getting Started In…”: A Series Not to Miss

**DOI:** 10.1371/journal.pcbi.0030224

**Published:** 2007-10-26

**Authors:** Olga Troyanskaya

In recent decades, computational biology has established itself as a critical part of biomedical research and as an ever-growing and highly interdisciplinary field. It seems that every year, new areas of research appear—sequence analysis now includes SNP detection and whole genome alignments, gene expression microarray data can be analyzed in concert with interaction networks, and protein structures can be used to predict potential drug targets. This diversity and development of bioinformatics has led to an increasing number of approaches and techniques that require understanding of areas as diverse as biology and computer science, medicine and statistics, chemistry and applied mathematics. With the knowledge required to explore each area of computational biology, the barrier grows for those trying to enter it. So how can one start learning about tiling array analysis or text mining in biology? What should a new graduate student read before analyzing his first microarray? How can a machine learning researcher find out what interesting problems in bioinformatics she may address with probabilistic graphical models? We hope that help is on the way.

This month, *PLoS Computational Biology* and the International Society for Computational Biology begin a series of short, practical articles for students and active researchers who want to learn more about new areas of computational biology and are unsure where or how to start. The aim of each article in the “Getting Started in…” series is to introduce the essentials: define the area and what it is about, highlight the debates and issues of relevance, and provide directions to the most relevant books, articles, or Web sites to find out more. The series will not include review articles or detailed tutorials; these are available in the Education section of the Journal. Rather, each “Getting Started in…” article will aim to be a cache of “go to” information for someone for whom the field is completely new. We hope each part of the series, written by experts in areas as diverse as data integration and phylogeny reconstruction, will be as invaluable as receiving an e-mail from a colleague who takes time and thought to offer the best advice and the essential introduction to his or her area of research.

The first expert to inform, motivate, and inspire readers to consider a new direction is Dr. Xiaole Shirley Liu, who introduces tiling microarrays. As the series progresses, we can look forward to learning about text mining and probabilistic graphical models, to name a few topics. We hope you find this new series useful and enjoyable. 

